# Evaluation of alcohol use behavior among patients cured through HCV elimination program in Georgia

**DOI:** 10.1186/s13104-024-06814-8

**Published:** 2024-06-10

**Authors:** Maia Butsashvili, Lasha Gulbiani, Giorgi Kanchelashvili, Tina Kamkamidze, Maia Kajaia, Salome Gudavadze, George Kamkamidze

**Affiliations:** 1 Health Research Union, 8 Nutsubidze str. Tbilisi, Tbilisi, 0171 Georgia; 2 University of Georgia, 77a M. Kostava str. Tbilisi, Tbilisi, 0171 Georgia

**Keywords:** HCV, SVR, Alcohol, Fibrosis

## Abstract

**Objective:**

The objective of the study was to understand the role of self-reported drinking behavior on liver health after achieving sustained viral response (SVR) among HCV patients.

**Results:**

The study was conducted in HCV treatment provider clinics in three cities in Georgia: Tbilisi, Batumi, and Telavi. Face-to-face interviews were conducted using a questionnaire developed specifically for this study. 9.5% considered themselves heavy drinkers, while 94.2% were aware that heavy alcohol consumption can progress liver fibrosis. During treatment, 97.8% abstained from alcohol, while 76.6% reported resuming drinking after achieving SVR. Additionally, 52.1% believed that moderate alcohol intake is normal for individuals with low fibrosis scores. Liver fibrosis improvement was more prevalent among individuals who abstained from alcohol after HCV diagnosis (85.4% vs. 71.4%, *p* < 0.01) and after achieving SVR (87.5% vs. 74.7% of those who resumed drinking after achieving SVR, *p* < 0.02). In conclusion, the majority of HCV patients abstain from alcohol during treatment but resume drinking after achieving SVR. Those who abstain from alcohol intake after HCV cure have a higher chance of liver fibrosis improvement.

**Supplementary Information:**

The online version contains supplementary material available at 10.1186/s13104-024-06814-8.

## Introduction

Alcohol is a major risk factor for liver disease, and exacerbates liver damage among patients with chronic viral hepatitis [1, 2]. Eastern Europe is one of the excessive alcohol drinking regions in the world, including Georgia. This is a wine producing country where vineyard ownership and producing homemade wine is very common, particularly in eastern part of the country [3].

Hepatitis C virus (HCV) infection is highly prevalent in Georgia. According to a national survey conducted in 2015, 5.4% of the general adult population of Georgia had chronic HCV infection (HCV RNA positive) [4, 5]. In April 2015, Georgia implemented a national HCV elimination program, with direct acting antiviral medications (DAAs) available free of charge for all patients enrolled in the program. By the end of 2021, more than 76,000 patients were treated through the HCV elimination program, and of those tested for cure, 98.9% achieved sustained viral response (SVR) [5, 6].

There is scarcity of published data on the role of alcohol on liver inflammation and fibrosis progression as well as on the knowledge of alcohol impact on liver health among HCV patients in Georgia. Understanding the role of drinking behavior on liver health after achieving SVR is important to plan and implement targeted interventions among patients cured of HCV to prevent progression of liver fibrosis.

This study aimed to evaluate the association of self-reported alcohol use with liver fibrosis level and alcohol consumption practice after achieving SVR.

## Main text

### Methods

#### The study sites

The study was conducted in three cities of Georgia (Tbilisi – the capital, Batumi in Western Georgia and Telavi in Eastern Georgia). One private outpatient clinic providing HCV diagnostics and treatment within HCV elimination program was selected in each city. Data were collected from October to December, 2020.

#### Study subjects

Patients treated through the HCV elimination program with DAAs and achieving SVR were randomly selected and invited to participate by a research assistant. SVR was determined by HCV PCR 12–24 weeks after completion of the treatment according to HCV elimination program guideline [6]. Patients achieving SVR earlier than 12 months prior to interview were excluded from the study. Among those willing to participate informed consent was obtained. Face-to-face interviews using a questionnaire developed by the research team for this study were conducted by trained interviewers (see uploaded survey tool in supplementary materials). Eight questions were used to collect socio-demographic data of survey participants. AUDIT tool was used to develop alcohol consumption questions in context of HCV treatment [7]. Few questions were added to understand survey respondents’ knowledge, attitude and practice towards alcohol intake before, during and after HCV treatment enrollment. All participants were ≥ 18 years of age.

Patients’ demographic and clinical data, including level of liver fibrosis, were extracted from the medical charts at the participating clinics. The HCV elimination program uses FIB-4 score (based on ALT, AST, age and platelet count) to assess liver fibrosis level, and liver elastography is performed for patients with a FIB-4 score of 1.46–3.24. Among the study participants, a follow-up evaluation of liver fibrosis was done to compare with baseline level (upon enrollment in the HCV treatment program). The same approach of follow-up liver fibrosis evaluation was made. Any decrease in liver fibrosis level (in kilopascals by liver elastography or FIB-4 score) was considered as an improvement [8]. Frequent alcohol consumption was defined as drinking alcohol nearly every day or 2–3 times a week [9].

Systematic visits were conducted at the study sites by the field manager. Double entry was done to ensure the accuracy of data entered from questionnaires into the database.

### Statistical analysis

Data analysis was conducted using the statistical package IBM SPSS 26.0. Pearson’s chi-square test and Fisher’s exact test, for small samples, were used to study the associations between categorical variables, with a *p*-value less than 0.05 considered statistically significant. Odds ratios (OR) and 95% confident intervals (95% CI) were calculated.

## Results

Overall, 500 patients were invited to participate in the survey, of whom 12.4% (*n* = 62) refused. Out of 438 individuals enrolled 82.9% (*n* = 363) were male, 9.6% (*n* = 42) were ≤ 35 years old, 98.6% (*n* = 423) were Georgian and 70.5% (*n* = 309) were married (Table 1). Over a third (35.9%, *n* = 153) of participants were unemployed and 36.3% (*n* = 159) had university degree. Regionally, 37.0% (*n* = 162), 36.6% (*n* = 159) and 26.9% (*n* = 117) of respondents lived in Tbilisi, Batumi and Telavi, respectively.

**Table 1:** Socio-demographic characteristics

Characteristic	Number of participants	Percentage
	N	%
**Gender**		
Male	363	82.9
Female	75	17.1
**Age**		
≤35	42	9.6
>35	396	90.4
**Ethnicity**		
Georgian	423	98.6
Other	6	1.4
**Marital status**		
Married	309	70.5
Not married	99	22.6
Other	30	6.8
**Education level**		
Vocational/High School	279	63.7
University	159	36.3
**Employment**		
Employed/Self employed	267	62.7
Unemployed	153	35.9
Retired	6	1.4
**Place of residence**		
Tbilisi	162	37.0
Batumi	159	36.6
Telavi	117	26.9

Attitudes towards alcohol consumption were evaluated among the survey participants. The majority (94.5%, *n* = 414) had used alcohol during their lifetime, of whom 9.4% (*n* = 39) considered themselves as heavy drinkers and 4.3% (*n* = 18) consumed alcohol nearly every day. For 10.8% (*n* = 42) of respondents > 10 drinks on one occasion was a normal amount of alcohol intake. Among those not considering themselves as heavy drinkers, 37.2% (*n* = 126) reported consuming > 5 drinks on at least one occasion.

The majority of respondents reported drinking wine most frequently (68.9%, *n* = 279), followed by vodka (12.6%, *n* = 51) and beer (5.2%, *n* = 21). When asked “usually, what is your reason to drink alcohol?” a plurality (37.9%, *n* = 150) responded “holidays”, followed by “to have fun” (36.4%, *n* = 144) and peer pressure (18.2%, *n* = 72).

Most (90.5%, *n* = 372) study subjects had at least one episode of getting drunk before their HCV diagnosis. The majority of study individuals (94.2%, *n* = 390) correctly identified “accelerated liver fibrosis” as a possible consequence of heavy alcohol drinking.

Among those who’d ever used alcohol, 34.1% (*n* = 141) completely avoided drinking alcohol after HCV diagnosis. More than half of participants (55.5%, *n* = 228) significantly reduced the amount of alcohol consumption after HCV diagnosis. The majority of participants (97.8%, *n* = 405) avoided drinking alcohol while taking HCV antiviral medications.

After achieving SVR, 76.6% (*n* = 315) reported that they consumed alcohol and 50.0% (*n* = 84) had at least one episode of getting drunk. For 10.9% (*n* = 45) of individuals, drinking alcohol was extremely unacceptable after cure from HCV, 76.6% (*n* = 315) declared that alcohol consumption is safe depending on liver fibrosis level, and 9.5% (*n* = 39) thought that drinking is acceptable regardless of fibrosis level (Table 2).

**Table 2:** Knowledge, Attitude and practice towards alcohol consumption

Characteristic	Number of participants	Percentage
	N	%
**Have you ever used alcohol?**		
Yes	414	94.5
No	24	5.5
**Do you consider yourself as a heavy drinker?**		
Yes	39	9.5
No/I don’t know	372	90.5
**What type of alcohol do you usually consume?**		
Wine	279	68.9
Vodka/chacha	51	12.6
Beer	21	5.2
Whiskey	6	1.5
Cognac	15	3.7
Other	3	0.7
All	30	7.4
**How often do you consume alcohol?**		
Nearly every day	18	4.5
2–3 times in a week	39	9.8
2–3 times in a month	57	14.4
Once in a month	159	40.2
1–2 times in a year	123	31.1
**In your opinion, what is your normal amount of alcohol intake on one occasion?**		
1–2 glasses	120	30.8
3–5 glasses	159	40.8
6–10 glasses	69	17.7
>10 glasses	42	10.8
**Usually, what is your reason for drinking alcohol?**		
To get drunk	21	5.3
Peer pressure	72	18.2
Holidays	150	37.9
To have fun	144	36.4
To cope with stress	6	1.5
Other	3	0.8
**Had you ever got drunk before HCV diagnosis?**		
Yes	372	90.5
No	39	9.5
**How many times a month did you usually get drunk, before HCV diagnosis?**		
Did not get drunk	12	4.3
1–2 times	69	24.7
3–5 times	33	11.8
6–10	12	4.3
>10 times	30	10.8
I don’t remember	123	44.1
**Heavy alcohol use can cause**:		
Chronic hepatitis C	3	0.7
Accelerate fibrosis	390	94.2
None	9	2.2
I don’t know	12	2.9
**Can heavy alcohol consumption cause liver fibrosis among individuals without HCV?**		
Yes	381	92.7
No	15	3.6
I don’t know	15	3.6
**Did you avoid drinking alcohol after HCV diagnosis?**		
Yes, completely	141	34.1
Yes, partially	267	64.5
No	6	1.4
**How many times did you get drunk after HCV diagnosis?**		
Didn’t get drunk	99	30.3
1–2 times	63	19.3
3–5 times	39	11.9
6–10 times	12	3.7
>10 times	21	6.4
I don’t remember	93	28.4
**Did you reduce the amount of alcohol intake after HCV diagnosis?**		
Yes, significantly	228	55.5
Yes, moderately	171	41.6
No	12	2.9
**Did you use alcohol while taking HCV antiviral medications?**		
Rarely	9	2.2
Never	405	97.8
**Have you used alcohol after cure from HCV?**		
Yes	315	76.6
No	96	23.4
**Have you ever got drunk after cure from HCV?**		
Yes	84	50.0
No	84	50.0
**How many times did you get drunk after cure from HCV?**		
1–2 times	48	51.6
1–2 times a month	30	32.3
1 time a week	12	12.9
Several times a week	3	3.2
**In your opinion, is it safe to use alcohol after cure from chronic hepatitis C?**		
Extremely unacceptable	45	10.9
Depends on fibrosis level	315	76.6
Acceptable regardless fibrosis level	39	9.5
I don’t know	12	2.9

### Association of alcohol use behavior with demographics

None of the patients aged ≤ 35 years reported drinking often, as opposed to 15.8% of those aged > 35 years (*p* < 0.01).

Among males, 12.5% thought that > 10 drinks in a single sitting was a normal amount of alcohol for health. For 77.8% of females and 23.2% of males 1–2 drinks on one occasion is normal amount of alcohol intake (OR = 2.12; 95% CI: 1.26–3.57).

Unmarried individuals were more likely to consider themselves as heavy drinkers compared to married people (13.3% vs. 9.2%, *p* = 0.11). Similarly, people who were not married were more likely to drink alcohol after achieving SVR (80.0%, *n* = 72), followed by married (77.6%, *n* = 228) and other marital status (55.6%, *n* = 15) participants (*p* < 0.05).

People who had higher educational level were more likely to know that accelerated fibrosis is a possible impact of heavy drinking; 97.9% (*n* = 141) of participants with university degree answered correctly, compared to 95.5% (*n* = 63) and 89.7% (*n* = 183) of participants with vocational and high school education, respectively (*p* < 0.01).

Gender and education level were associated with changing behavior towards drinking alcohol. After HCV diagnosis, 82.4% of females vs. 27.5% of males refrained to drink alcohol (*p* < 0.001). Males were also more likely to consume alcohol after achieving SVR compared to females (81.5% vs. 44.4%; OR = 5.51; 95% CI: 3.02–10.03). Among respondents with a high school or university degree, 37.3% and 37.5% respectively abstained alcohol intake after HCV diagnosis while only 18.2% of individuals with less educational level did so (*p* < 0.05).

Half (51.3%, *n* = 183) of male respondents reduced alcohol consumption significantly after HCV diagnosis compared to 83.3% (*n* = 45) of females (*p* < 0.001).

The proportion of participants having at least one occasion of drinking alcohol while taking HCV medications was higher among ≤ 35 years old individuals (16.7% vs. 0.8%; *p* < 0.001).

The proportion of patients who had improved liver fibrosis was significantly higher among those who drank rarely (80.0% vs. 36.4%. OR = 0.14; 95% CI: 0.06–0.30) and those who consumed ≤ 5 drinks on one occasion (81.0% vs. 66.7%. OR = 2.12. 95% CI: 1.26–3.57). Liver fibrosis improvement was also more likely among those who avoided drinking alcohol after HCV diagnosis vs. those who continued to drink (85.4% vs. 71.4%; OR = 2.33; 95% CI: 1.30–4.17). Patients who did not use alcohol after cure from HCV had higher rate of liver fibrosis improvement compared to those who consumed alcohol after achieving SVR (87.5% vs. 74.7%. OR:0.42. 95% CI: 0.19–0.89) (Table 3).

**Table 3:** Drinking behavior by liver fibrosis improvement

Characteristics	Liver fibrosis improved		Liver fibrosis not improve		OR; 95% CI
	*N*	%	*N*	%	
**Have you ever used alcohol?**					
Yes	258	76.8	78	23.2	3.30 (1.03–10.5) 1
No	6	50.0	6	50.0	
**Do you consider yourself as a heavy drinker?**					
Yes	21	70.0	9	30.0	0.68 (0.30–1.57) 1
No/DK	234	77.2	63	22.8	
**Frequency of alcohol consumption**					
Often	12	36.4	21	63.6	0.14 (0.66–0.30) 1
Rare	228	80.0	57	20.0	
**Usually, how many drinks do you have during one occasion?**					
1–5 glasses	153	81.0	36	19.0	2.12 (1.26–3.57) 1
>5 glasses	84	66.7	42	33.3	
**Had you ever got drunk before HCV diagnosis?**					
Yes	225	75.8	72	24.2	0.62 (0.25–1.56) 1
No	30	83.3	6	16.7	
**Can alcohol intake cause liver damage among patients without HCV?**					
Yes	252	78.5	69	21.5	5.47 (1.88–15.9) 1
No/DK	6	40.0	9	60.0	
**Did you avoid drinking alcohol after HCV diagnosis?**					
Yes	105	85.4	18	14.6	2.33 (1.30–4.17) 1
No	150	71.4	60	28.6	
**Did you reduce alcohol consumption after HCV diagnosis?**					
Yes	249	77.6	72	22.4	3.4 (1.08–11.04) 1
No	6	50.0	6	50.0	
**Did you use alcohol while taking HCV antiviral medications?**					
Yes	6	100	0	0.0	*
No	252	76.4	78	23.6	
**Have you used alcohol after cure from HCV?**					
Yes	195	74.7	66	25.3	0.42 (0.19–0.89) 1
No	63	87.5	9	12.5	
**Did you get drunk after cure from HCV?**					
Yes	36	63.2	21	36.8	0.45 (0.19–1.05) 1
No	45	78.9	12	21.1	

* Fisher’s exact test *p* *= 0.4. Confidence intervals could not be calculated*

## Discussion

This is the first study evaluating alcohol use behavior among persons cured of hepatitis C through the HCV elimination program in Georgia. As producing alcoholic beverages is part of Georgian culture and alcohol consumption (especially wine) is very common, understanding the role of alcohol in the progression of liver disease among people cured from HCV infection is important.

The majority of survey participants knew that heavy drinking can accelerate liver fibrosis. However, despite having an adequate understanding of the harmful effects of alcohol on liver health, 77.1% (*n* = 296) had consumed alcohol after achieving SVR, and 50.1% (*n* = 77) had experienced at least one episode of drunkenness after SVR. Thus, it is very important to include the message on the harmful effect of alcohol on liver health even after achieving SVR in physicians’ consultations among HCV patients.

Our study revealed the harmful effect of alcohol on liver health. According to our findings people not drinking alcohol after HCV diagnosis had 2.5 times higher chance of improvement of liver fibrosis level compared to drinkers (71.4% vs. 28.6%). The finding is consistent with many published studies on the harmful effects of alcohol on liver health [1, 10, 11].

A study in the United States also found a discrepancy between the knowledge of harmful effects of alcohol and alcohol consumption, where 84% of respondents said that drinking alcohol was not safe for HCV infected persons, but still 62% reported moderate use and 11% had “at risk” use (more than 14 drinks per week for males and more than 7 drinks per week for females) [12].

Overall, alcohol consumption after knowing HCV status decreased among participants in the current study; 97.1% reduced alcohol intake after HCV diagnosis, 97.8% never drank while taking HCV treatment medications, but most (76.6%) of those patients who stopped drinking during the treatment resumed alcohol consumption after achieving SVR, with 50.0% having at least one episode of getting drunk after HCV treatment. This is similar to another study conducted in USA where decrease of alcohol amount was observed during and after HCV treatment. Alcohol consumption improved during DAA treatment and was sustained after completing the course (< 12 weeks before treatment 32.5 g/day; during treatment 20.0 g/day; and 12–24 weeks after treatment 23.7 g/day) [13].

People who were not married were more likely to drink alcohol after achieving SVR showing that support from family members can play a positive role to prevent alcohol consumption after successful treatment.

Despite promising findings about attitudes toward alcohol consumption, resuming to drink after achieving SVR remains a public health challenge.

## Conclusion

In conclusion, drinking alcohol is common in Georgia and a substantial proportion of people in the HCV treatment program consume alcohol. Abstaining from alcohol is advantageous to improvement in fibrosis, even after SVR has been achieved. Although the majority of HCV patients in this study did not drink alcohol during treatment, most resumed drinking after achieving SVR. These findings present an opportunity to focus messaging and education for patients during DAA treatment to improve outcomes even after completion of treatment.

## Limitations

One of the limitations of this study is self-reported alcohol consumption and retrospectively collected data, which could result in some information bias. Also, we did not use an internationally recognized survey instrument for alcohol related KAP; the questionnaire was created for this study, and the survey tool was piloted before the survey.

### Electronic supplementary material

Below is the link to the electronic supplementary material.

Supplementary Material 1

## Data Availability

Data will be available upon request. The corresponding author will be responsible for providing access to any requested data. Corresponding author’s email: lashagulbiani7@gmail.com.

## Abbreviations

HCV: Hepatitis C virus
DAAs: Direct acting antiviral medications
SVR: Sustained viral response
OR: Odds ratio
