# Supplementary material for: Evaluation of alcohol use behavior among patients cured through HCV elimination program in Georgia
Source: BMC Res Notes. 2024 Jun 10;17:160. doi: 10.1186/s13104-024-06814-8 (PMC11165773; doi:10.1186/s13104-024-06814-8)
Supplement: Supplementary file 1 — Supplementary Material 1 [file 13104_2024_6814_MOESM1_ESM.pdf]

Evaluation of alcohol use behavior among patients cured through HCV elimination program in Georgia

**Liver assessment** (filled by interviewer)	
Pretreatment fibrosis level (by fibroscan kPa or FIB4 score)	______________________________
Posttreatment fibrosis level (by fibroscan kPa or FIB4 score)	______________________________

Questionnaire

Section 1. Socio-demographic characteristics

N	Question	Answer
1	Age	_____ years
2	Gender	1. MaleFemale
3	Nationality	1. Georgian<br>2. Other *(Please clarify_______________________)*
4	Marital status	1. Married<br>2. Single<br>3. Other (___________________)
5	Number of family members	______ Members
6	Education level	1. Secondary<br>2. Vocational<br>3. High school<br>4. Bachelor<br>5. Master/PhD
7	Employment status	1. Employed/self employed<br>2. Not employed<br>3. Student<br>4. Retired<br>5. Cannot work<br>6. Other *(Please clarify_______________________)*

8	Place of residence	1. Tbilisi<br>2. Batumi<br>3. Telavi

Section 2. Knowledge and use of alcohol

9	Have you ever used alcohol?	1. Yes<br>2. No **(end of interview)**
10	Do you consider yourself as a heavy drinker?	1. Yes<br>2. No<br>3. I don't know
11	How many years of experience of drinking alcohol do you have?	_____ years
12	What type of alcohol do you usually consume?<br><br>*(Mark all that applies)*	1. Wine<br>2. Vodka/Chacha<br>3. Bear<br>4. Whiskey<br>5. Cognac<br>6. Other *(please specify______________________)*
13	How often do you consume alcohol?	1. Almost every day<br>2. 2-3 times a week<br>3. 2-3 times a month<br>4. Once a month<br>5. 1-2 times a year
14	Usually, how many drinks do you have during one drinking time?<br><br>*(Mark all that applies)*	1. 1-2 glassess of ________________________<br>2. 3-5 glassess of ________________________<br>3. 6-10 glassess of _______________________<br>4. More than 10 glassess of __________________
15	In your opinion, what is the normal amount of alcohol intake on one occasion?<br><br>*(Mark all that applies)*	1. 1-2 glassess of ________________________<br>2. 3-5 glassess of ________________________<br>3. 6-10 glassess of _______________________<br>4. More than 10 glassess of __________________

16	Usually, what is the reason of drinking alcohol?	1. To get drunk<br>2. Mostly, I have to drink against my willing<br>3. Celebrating<br>4. To have fun<br>5. To escape pain/sadness<br>6. Other *(please specify____)*
17	Had you ever got drunk before HCV diagnosis?	1. Yes<br>2. No **(Skip to question #19)**
18	How many times a month did you usually get drunk, before HCV diagnosis?	1. Never<br>2. 1-2 times<br>3. 3-5 times<br>4. 6-10 times<br>5. More than 10 times<br>6. Don't remember
19	Heavy alcohol use can cause:	1. Chronic hepatitis C<br>2. Accelerate liver fibrosis process<br>3. None of abovementioned<br>4. I don't know
20	Can heavy alcohol consumption cause liver fibrosis among individuals without HCV?	1. Yes<br>2. No<br>3. I don't know
21	Did you avoid to drink alcohol after HCV diagnosis?	1. Yes, completely **(Skip to question #23)**<br>2. Yes, partially<br>3. No
22	How many times did you get drunk after HCV diagnosis?	1. Never<br>2. 1-2 times<br>3. 3-5 times<br>4. 6-10 times<br>5. More than 10 times<br>6. I don't remember
23	Did you reduce the amount of alcohol intake after HCV diagnosis?	1. Yes, significantly<br>2. Yes, moderately<br>3. No
24	Did you use alcohol while taking HCV antiviral medications?	1. Yes, many times<br>2. Rarely<br>3. Never<br>4. I don't remember

25	Have you used alcohol after cure from HCV?	1. Yes<br>2. No **(Skip to question #28)**
26	Have you ever got drunk after cure from HCV?	1. Yes<br>2. No **(Skip to question #28)**
27	How many times did you get drunk after cure from HCV?	1. 1-2 times<br>2. I get drunk 1-2 times a month<br>3. I get drunk once a week<br>4. I get drunk several times a week
28	In your opinion, is it safe to use alcohol after cure from chronic hepatitis C?	1. Absolutely not<br>2. It is safe, if low level of liver fibrosis<br>3. It isn't safe if high level of liver fibrosis<br>4. It is safe regardless the level of liver fibrosis<br>5. I don't know
